# Increased ACh-Associated Immunoreactivity in Autonomic Centers in PTZ Kindling Model of Epilepsy

**DOI:** 10.3390/biomedicines8050113

**Published:** 2020-05-08

**Authors:** Enes Akyüz, Züleyha Doğanyiğit, Yam Nath Paudel, Emin Kaymak, Seher Yilmaz, Arda Uner, Mohd. Farooq Shaikh

**Affiliations:** 1 Department of Biophysics, Faculty of Medicine, Yozgat Bozok University, Yozgat 66100, Turkey; 2 Department of Histology and Embryology, Faculty of Medicine, Yozgat Bozok University, Yozgat 66100, Turkey; zuleyha.doganyigit@yobu.edu.tr (Z.D.); emin.kaymak@yobu.edu.tr (E.K.); 3 Neuropharmacology Research Strength, Jeffrey Cheah School of Medicine and Health Sciences, Monash University Malaysia, Bandar Sunway 47500, Selangor, Malaysia; yam.paudel@monash.edu; 4 Department of Anatomy, Faculty of Medicine, Yozgat Bozok University, Yozgat 66100, Turkey; seher.yilmaz@yobu.edu.tr; 5 Faculty of Medicine, Yozgat Bozok University, Yozgat 66100, Turkey; ardauner5@gmail.com

**Keywords:** acetylcholine, Kir channels, M2 receptors, epilepsy

## Abstract

Experimental and clinical studies of cardiac pathology associated with epilepsy have demonstrated an impact on the autonomic nervous system (ANS). However, the underlying molecular mechanism has not been fully elucidated. Molecular investigation of the neurotransmitters related receptor and ion channel directing ANS might help in understanding the associated mechanism. In this paper, we investigated the role of acetylcholine (ACh), which demonstrates both sympathetic and parasympathetic roles in targeted expression in terms of the relevant receptor and ion channel. Inwardly rectifying potassium (Kir) channels play a significant role in maintaining the resting membrane potential and controlling cell excitability and are prominently expressed in both the excitable and non-excitable tissues. The immunoreactivity of ACh-activated Kir3.1 channel and muscarinic ACh receptors (M2) in autonomic centers such as the brainstem, vagus nerve (VN) and atria of heart was confirmed by both histological staining and pathological tissue analysis. Significant upregulations of Kir3.1 and M2 receptors were observed in pentylenetetrazol (PTZ)-kindled epileptic rats for all related tissues investigated, whereas no pathological difference was observed. These findings provide proof-of-concept that changes in ACh-associated immunoreactivity might be linked to the ANS dysfunctions associated with epilepsy.

## 1. Introduction

Epilepsy is a devastating disorder of the central nervous system (CNS) caused by an imbalance in excitation and inhibition, which might be due to functional and structural changes in the brain. However, despite extensive development in pre-clinical and clinical research, the precise mechanism underlying the pathogenesis of seizure generation is not fully known. Epilepsy is a chronic neurological disease is associated with a 24-fold higher risk of sudden unexpected death compared to the normal population, reflecting a further need to unravel the relationship between the brain and autonomic nervous system (ANS) [1]. New treatment methods should be sought, especially for drug-resistant cases, by enlightening the entire molecular mechanism centered on the vagus nerve (VN) that provides the link between the brain and the heart.

In the case of drug-resistant young adult, vagus nerve stimulation (VNS) is used as a treatment method that has been reported to suppress epileptic seizures [2,3]. The VN and brainstem are considered as autonomic targets in terms of sympathetic/parasympathetic effects, both to decrease seizures and to suppress the effect of recent seizures on the ANS. Seizures also alter the respiratory system and heart function depending on the ANS dysfunction [4]. Accordingly, patients with epilepsy frequently have interictal autonomic dysfunction, while several studies report that heart rate variability (HRV) abnormalities, including generalized tonic–clonic seizures, are affected [5,6,7]. Molecular investigation of the neurotransmitters and related ion channels that direct HRV helps to clarify the basic illumination of the mechanism as well as new treatment options. 

The parasympathetic stimulation effects of the ANS might be listed briefly in a variety of physiological features such as constriction in the eye muscles, a decrease in the cardiac contraction, heart rate, and dilatation in the coronary vessels. Of these features, controlling the contraction mechanism of the cardiomyocyte is provided by the VN acting as a “bridge” between the brain and the heart. The 10th cranial nerve, the VN, consists of 80% afferent and 20% efferent fibers that provide sensory and motor innervation [8]. The afferent fibers are targeted for therapy through stimulation in drug-resistant epileptic cases.

The release of acetylcholine (ACh) in the hippocampus is an essential target for learning and memory. Cholinergic dysfunction in this region has been associated with epilepsy, Alzheimer’s disease, schizophrenia and various neurological diseases [9]. For ANS, the VN releases ACh, allowing the body to be soothed with a parasympathetic effect.

Simultaneous impairment in consciousness and autonomic control indicate a pathological process in the brainstem. The inclusion of the brainstem in events leading to sudden unexpected death in epilepsy (SUDEP) is also supported and expanded by findings in SUDEP animal models [10]. It is known that respiratory/heart functions are controlled by the coordination of the sympathetic and parasympathetic system. Therefore, besides ACh, sympathetic projections of the brainstem, noradrenaline and adrenergic neurons are thought to play a role in the mechanism of SUDEP [11]. In the SUDEP animal model induced with “audiogenic” seizures in Wistar rats, ß-adrenergic blockers have been reported to reduce sympathetic nervous system-related cardiac pathologies and might be useful for SUDEP [12,13]. In addition, increased levels of adrenaline and noradrenaline due to sympathetic activation and increased heart rate/blood pressure processes might play a role in SUDEP pathophysiology [14]. The regions highlighted by the studies include major serotoninergic, noradrenergic and cholinergic centers of the brainstem, which have broad and effective afferent connections not only among themselves, but also with cortical/subcortical structures [15]. Thus, the expression of ACh-related receptors and ion channels, which represent the cholinergic center in the brainstem, must be evaluated in terms of ANS pathology in epilepsy.

Inwardly rectifying potassium channels (Kir channels) demonstrate essential functions in excitable structures such as neurons, cardiomyocytes or skeletal muscle cells, working towards the end of the action potential and performing their physiological roles by operating like a diode, transferring K^+^ ions into the cell [16]. ACh actions are carried out by ACh-activated muscarinic potassium channels. The channels are activated via the M2 muscarinic receptor to form a heterotetramer with Kir3.1 channels [17]. Muscarinic acetylcholine receptor M2, also known as cholinergic receptor, has the function of reducing the heart rate to normal sinus rhythm due to the stimulating effects of the parasympathetic nervous system. The acceptance of heart rate variability as a biomarker in cardiac problems accompanying epilepsy [18] has paved the way for the research of M2 receptors in this study.

The Kir3 channel is stimulated by various neurotransmitters such as ACh, dopamine, opioids, serotonin, somatostatin, adenosine and gamma aminobutyric acid (GABA) [19]. Kir3 channels function through G-proteins. For this reason, Kir3 channels are also called G-protein-linked inwardly rectifying potassium channels (GIRK). The appropriate signaling molecule provides stimulation to the G-protein bound receptors (GPCR). Then, the appropriate subunit of the G-protein interacts with the Kir3 channel [20]. ACh released from the VN binds and activates M2 receptors in pacemaker cells located in the sinoatrial node. As a result of the binding, it provides the binding of GDP-bound G protein (Gαi (GDP) βγ). The activated receptor allows the removal of GDP from the G protein alpha subunit (Gai) that allows the GTP in the cell to bind. The GTP-bound Ga and G protein beta–gamma subunit (G βγ) is then separated from the receptor and, beta and gamma are also separated from each other. As a result, the G βγ subunit that is free on the membrane surface inside the cell is connected to the GIRK channel and causes it to open. The opened GIRK channel fulfills its duty in the action potential of the cardiac cell [21].

ACh-activated Kir3 channels and muscarinic ACh receptors (M2) that play a crucial role in ACh-mediated neurotransmission have been identified as candidates for autonomic involvement accompanying epilepsy [22]. The determination of the immunoreactivity of the ACh neurotransmitter released to the autonomic systems, such as the brainstem, VN, and heart might help to find new therapeutic centers for both the clarification of the molecular mechanism and VNS applied as a treatment.

Cholinergic neurons play different functional roles in the brainstem and peripheral nervous system. It has been shown that, in epileptogenesis, the cholinergic system, especially in the hippocampal region of the brain, undergoes structural and functional changes [23,24]. In addition, pedunculopontine, another primary cholinergic neuron source in the brainstem, has been shown to prevent cortical dysfunction during seizures by the selective activation of cholinergic neurons in the tegmental nucleus [25]. In addition, it has been reported that M1 muscarinic receptors in the medial septum region of the hippocampus integrate the inputs of vagal afferents from the brainstem into the hippocampus [26]. It is known that vagal messages are transmitted from the brainstem to the hippocampus, and it is hypothesized that the VN provides the connection between the brainstem and the peripheral systems [27]. Cholinergic neurons that are shown to be affected in the brain by epilepsy are also expected to be affected in the peripheral structure. For this reason, it is speculated that different parts could be sensitive to epilepsy.

In the light of the available literature, the current study aimed to elucidate the mechanism of epilepsy in the ANS by determining the relevant receptor and ion channel immunoreactivity in terms of dividing the VN into the thoracic/cervical parts, brainstem regions, and cardiac tissue. The hypothesis testing included the examination of the relationship between abnormal activity states in the brainstem, VN and heart that play a role in the regulation of autonomic control in epilepsy for the M2 receptor and Kir3.1 channel.

## 2. Material and Method

In the current experiment, Wistar albino rats (280–380 g, *n* = 34) were used from Kayseri Erciyes University Research Center. They were housed in a controlled environment at a temperature of 24 ± 2 °C and humidity of 60% under a 12-h light/dark cycle. Animals were given free access to water and standard food. All procedures were applied in strict accordance with the recommendations in the Guide for the Care and Use of Laboratory Animals adopted by the National Institutes of Health (USA) and the Declaration of Helsinki. The experimental protocol of this study was approved by the Animal Ethics Committee of Kayseri Erciyes University (ethics committee decision number: 2019/027). Rats were anesthetized with ketamine/xylazine (90/10 mg/kg, intraperitoneal (I.P.)) and all efforts were made to minimize animal suffering.

### 2.1. Experimental Epilepsy Model

A pentylenetetrazol (PTZ) kindling model of epilepsy was used and the experimental animals were differentiated into different groups (either control or PTZ-kindled groups).

#### 2.1.1. Control Groups 

A total of 0.5 cc of saline was given every two days. Male and female control groups (*n* = 7) were assigned as seven animals per experiment setup. Intraperitoneal (I.P.) saline was given to the groups to undergo equal injection treatment. 

#### 2.1.2. PTZ Kindling Groups (*n* = 10 per each)

Epileptic seizures were induced by periodic administration of PTZ (35 mg/kg, I.P.) for one month to develop kindling in the animals. PTZ (P6500, Sigma, St. Louis, MO, USA), a GABA_A_ receptor antagonist used in the model was dissolved in 0.9% NaCl solution and was prepared I.P. at a dose of 35 mg/kg. The solution was injected into the rats three days a week (Monday, Wednesday and Friday) for a month, and their behaviors were observed for 30 min individually post-injection, according to the earlier developed protocol [28] and epileptic seizure scoring was done as follows using the same protocol [28].

Phase 0: No response to PTZ;
Phase 1: Continuous ear and facial twitching;
Phase 2: Myoclonic body jerks;
Phase 3: Clonic forelimb convulsions;
Phase 4: Tonic–clonic seizures;
Phase 5: Generalized tonic–clonic seizures;
Phase 6: Death.

One week after the last PTZ injection (13th injection), high dose PTZ (50 mg/kg, I.P.) was given to animals to demonstrate improved seizure sensitivity in both female and male PTZ-kindled rats. Any animal with phase 4 or 5 seizures was considered to be completely kindled [29].

### 2.2. Dissection of VN

According to the Powley et al. study, after the application of ketamine/xylazine (90/10 mg/kg, I.P.) for anesthesia, the chest wall of the rats was cleaned with alcohol and the costa and sternum were removed by incision towards the sternum through the diaphragm [30]. Right and left VN was released and dissected in the cervical and thoracic region.

### 2.3. Histological and Pathological Staining

The brainstem, VN and heart tissue dissected for histological examination were immediately detected with a 4% formaldehyde solution. The detected tissues were then dehydrated by passing through a graded alcohol series. Tissues that were transparent with xylol were embedded in paraffin. hematoxylin–eosin (HE) staining was performed on 5–6-μm-thick sections taken from paraffin blocks and histopathological changes (in terms of cell shape, morphology, number, edema, etc.) in the heart tissue were determined by light microscopy.

**Figure 1:**
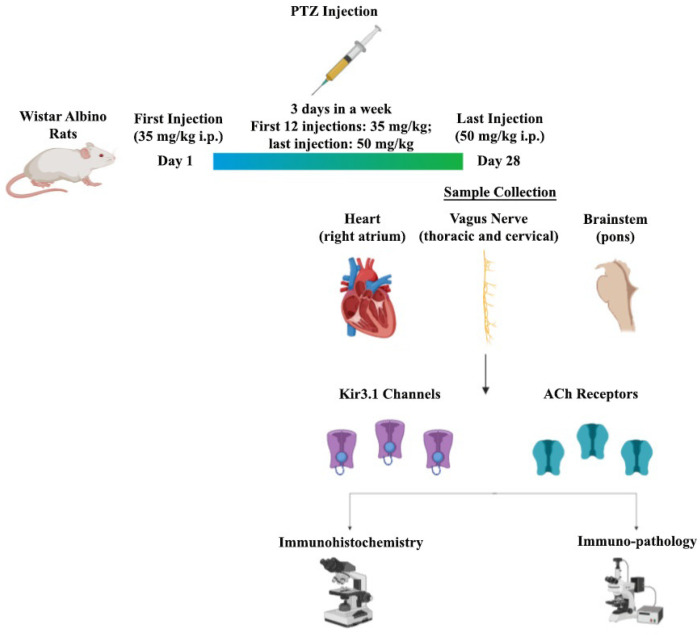
A pictorial representation of an experimental protocol.

Marking was done by an avidin–biotin–peroxidase method to determine the expression of the ACh-related ion channel and receptor differences in the brainstem, VN, and heart tissue. Sections 5–6 μm in size were taken and were kept at 60 °C overnight. They were firstly rehydrated by passing through xylene and then through a graded alcohol series, then washed three times for 5 min with phosphate buffer (PBS). Afterward, the sections boiled 3 × 5 times at 600 W in a microwave oven with 5% citrate buffer for antigen recovery and were kept in the same buffer solution for 20 min at room temperature. The sections washed again with PBS were treated with 3% hydrogen peroxide (H_2_O_2_) for 5 min to prevent endogenous peroxidase activity and the ABC staining system staining kit was used for the next steps. Block serum was applied at room temperature for 20 min to ensure that the areas outside the antigenic areas were closed in the sections washed again with PBS. Immediately, Kir3.1 (mouse monoclonal, 1:200, Alomone Labs, Jerusalem, Israel [APC-005]) and M2 receptor (rabbit polyclonal, 1:200, Alomone Labs, Jerusalem, Israel [APC-002]) primary antibodies were dropped and kept at +4 °C for one night and incubated the next day for 20 min. As a negative control, PBS was used instead of the primary antibody. After washing, the sections were incubated with a biotin secondary antibody for 30 min and then washing was repeated. The sections treated with the avidin–biotin (AB) enzyme reagent for 30 min were washed with the peroxidase substrate in the kit showing the diaminobenzidine (DAB) for 5 min; finally, they were washed with deionized H_2_O for 5 min. The sections opposite, stained with Gill’s hematoxylin, were washed several times with deionized H_2_O. As the last step, water was removed with increasing alcohol series and sections passed through xylene were covered with entellan. Images obtained with a DP71 digital camera under an Olympus BX51 model light microscope were evaluated in terms of expression differences in the Image-J software program. The entire experimental procedure is depicted in Figure 1.

PTZ (35 mg/kg, I.P.) was injected three times a week, continuing for 28 days, where the last injection of PTZ was with 50 mg/kg, I.P. The sample was collected at the end of an experiment, mainly from the brainstem, VN, and heart dissection, and was then immunohistochemically analyzed.

### 2.4. Statistical Analysis

The SPSS program was used for statistical analysis. Results are shown as the average standard error margin. The inter-group comparison was made with the one-way ANOVA test and the post-hoc Tukey test was used for binary comparisons. The statistical value of *p* < 0.05 was considered as significant.

## 3. Results

### 3.1. Scores of PTZ Kindling Epilepsy Model 

**Figure 2:**
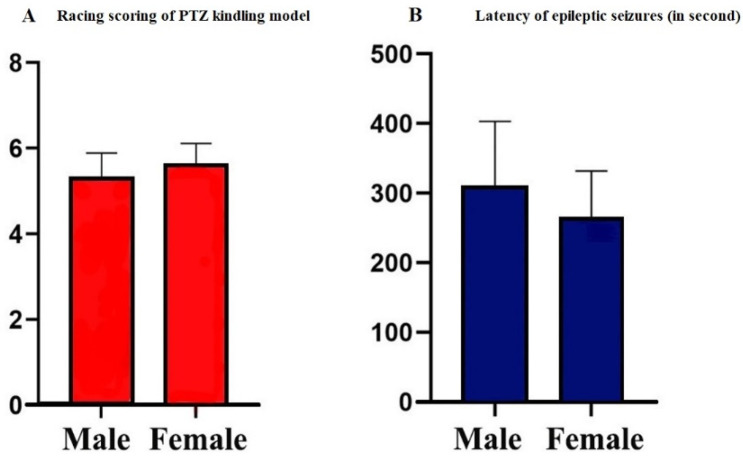
Racing score and seizure latency exhibited by male and female rats after each injection of pentylenetetrazol (PTZ). Values are presented as the mean ± SEM, *n* = 10 for each group. At the 13^th^ injection, generalized tonic–clonic seizures, corresponding to phase 5 of the Racine’s scaling system score, were observed in both male and female rats (5.31 ± 0.58 and 5.2 ± 0.47) (**A**). PTZ-kindled female rats had their first seizure in 266 ± 66 s while PTZ-kindled male rats had their first seizure in 308 ± 95 s (**B**).

After the PTZ treatment, kindled epileptic seizures were gradually induced. At the 13th injection, generalized tonic–clonic seizures, corresponding to phase 5 of the Racine’s scaling system score, were observed in both male and female rats (5.31 ± 0.58 and 5.2 ± 0.47, Figure 2A). Latency to first seizure was calculated for both groups. PTZ-kindled female rats had their first seizure in 266 ± 66 s, while PTZ-kindled male rats had their first seizure in 308 ± 95 s (Figure 2B). No significant changes in the body weights of rats were found during the PTZ injection process (data not shown).

### 3.2. Histopathological Evaluation 

**Figure 3:**
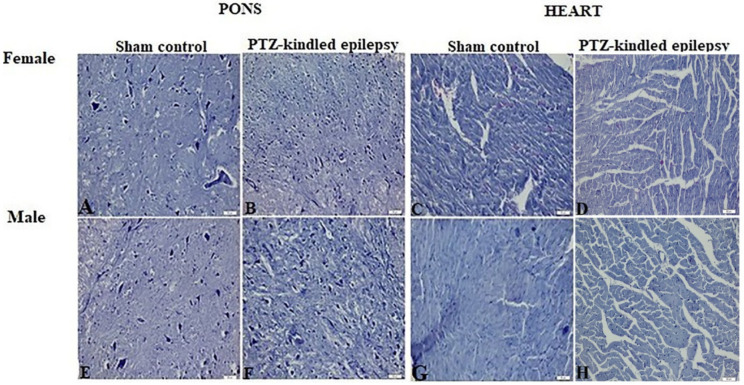
Representative HE staining images of the brainstem (pons) and heart (atria) sections stained with blue in sham control and PTZ-kindled epilepsy of rat model. For female; **A**: pons image of control groups, **B**: pons image of PTZ-kindled of epilepsy, **C**: heart image of control groups, **D**: heart image of PTZ-kindled epilepsy. For male; **E**: pons image of control groups, **F**: pons image of PTZ-kindled of epilepsy, **G**: heart image of control groups, **H**: heart image of PTZ-kindled epilepsy. Pictures were taken at the magnification of ×200. Scale bar: 50 μm.

**Figure 4:**
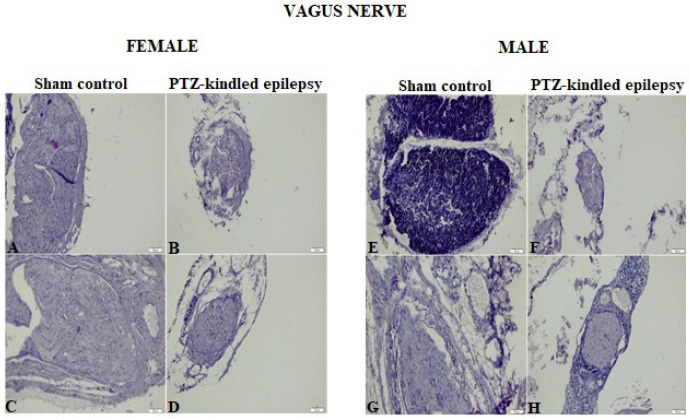
Representative HE staining images of VN sections stained with blue in sham control and PTZ-kindled epilepsy of rat model for male and female rats. For female; **A**: cervical VN image of control groups, **B**: thoracic VN image of control groups, **C**: Cervical VN image of PTZ-kindled epilepsy, **D**: Thoracic VN image of PTZ-kindled epilepsy. For male; **E**: cervical VN image of control groups, **F**: thoracic VN image of control groups, **G**: Cervical VN image of PTZ-kindled epilepsy, **H**: Thoracic VN image of PTZ-kindled epilepsy. Pictures were taken at a magnification of ×200. Scale bar: 50 μm.

Images obtained from HE staining procedure applied to the brainstem, heart tissue, and VN samples were represented in Figure 3 and Figure 4, respectively. Upon analysis of the tissue samples from the male and female rats, none of them showed a histologically significant difference between the experimental group and the control group. The absence of significant pathological findings in epileptic tissues is an indication that the model was created without mechanical damage such as hemorrhaging and tearing. 

### 3.3. Immunohistochemical Findings

Immunoreactivity results are explained under three different subtitles as the brainstem, VN and heart results.

#### 3.3.1. Brainstem

**Figure 5:**
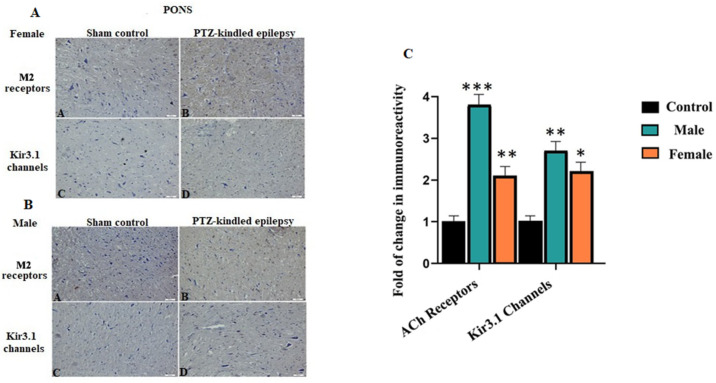
Examples of immunoreactivity for ACh receptors and Kir3.1 channel within the brainstem. Immunoreactivity was significantly increased for both ACh-associated centers in the represented images, the result for male rats was prepared. For female (**A**) and male (**B**) in terms of immunoreactivity at pons; **A**: Image of M2 receptors for control groups, **B**: Image of M2 receptors for PTZ-kindled of epilepsy, **C**: Image of Kir3.1 channels for control groups, **D**: Image of Kir3.1 channels of PTZ-kindled epilepsy. Pictures were taken at a magnification of ×200. Scale bar: 50 μm. Fold change in immunoreactivity for M2 receptors and Kir3.1 channel within the brainstem (**C**). For male, PTZ-kindled rats, 3.8-fold change in the M2 receptor and 2.7-fold change in Kir3.1 channel were observed when compared to the controls. For female PTZ-kindled rats, there was a 2.1-fold change in M2 receptors and 2.2-fold change in Kir3.1 channel when compared to the controls, * *p* < 0.05, ** *p* < 0.01, *** *p* < 0.001.

As a result of PTZ kindling, an immune histological staining analysis confirmed that the areas stained with related antibodies, the M2 receptor and Kir3.1 channel, were significantly immunoreactively increased in the pons of the brainstem compared to the normal control (Figure 5A,B). For female PTZ-kindled rats, there was a 2.1-fold change in M2 receptors and 2.2-fold change in Kir3.1 channel when compared to the controls. Moreover, for the male, PTZ-kindled rats, 3.8-fold change in the M2 receptor and 2.7-fold change in Kir3.1 channel were observed when compared to the controls, * *p* < 0.05, ** *p* < 0.05, and *** *p* < 0.05. All these fold changes are demonstrated in Figure 5C. Since the pons is the brainstem region closest to the VN, it has been identified as the main target. Because the activation of the channel and the functional operation of the receptor depend on the ACh neurotransmitter, the increased immunoreactivity of the Kir3.1 channel and M2 receptors in pons tissue indicates the evaluation of parasympathetic centers. 

#### 3.3.2. VN

**Figure 6:**
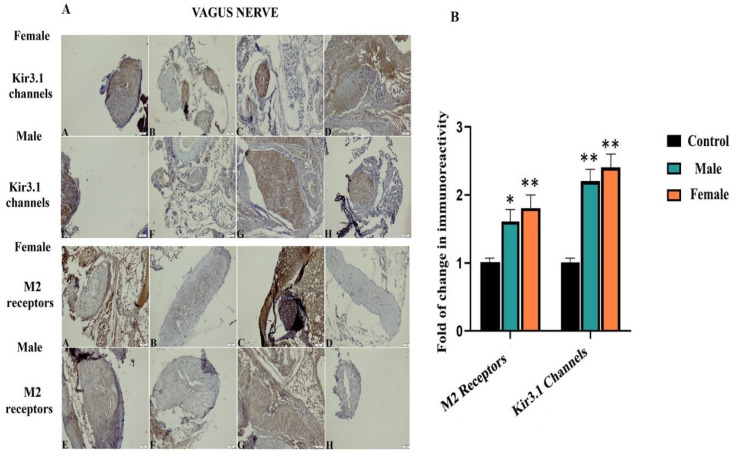
Immunoreactivity results of Kir3.1 channel and M2 receptors in the VN. In both groups, increased immunoreactivity in cervical VN was observed. For female groups; **A**: Image of cervical VN of control groups, **B**: Image of thoracic VN of control groups, **C**: Image of cervical VN of PTZ-kindled epilepsy, **D**: Image of thoracic VN of PTZ-kindled epilepsy. For male groups; **E**: Image of cervical VN of control groups, **F**: Image of thoracic VN of control groups, **G**: Image of cervical VN of PTZ-kindled epilepsy, **H**: Image of thoracic VN of PTZ-kindled epilepsy. Pictures were taken at a magnification of ×200. Scale bar: 50 μm (**A**). Fold change in immunoreactivity for M2 receptors and Kir3.1 channel in the VN (**B**). For cervical VN, in terms of the immunoreactivity of the M2 receptor, a 1.6-fold change in male and a 1.8-fold change in female rats compared to controls can be seen; for cervical VN, in terms of the immunoreactivity of Kir3.1, a 2.2-fold change in male and a 2.4-fold change in female rats compared to thoracic VN can be seen, * *p* < 0.05, ** *p* < 0.01.

We aimed to dissect the VN as two parts, in terms of thoracic and cervical sections. For this purpose, results were prepared separately for M2 receptors and the Kir3.1 channel, as demonstrated in Figure 6A. According to the results, the PTZ-kindled epilepsy of both rats (male and female) showed a significant immunoreactive increase within cervical VN, whereas thoracic VN showed no change. For cervical VN, in terms of the immunoreactivity of the M2 receptor, there was a 1.6-fold change in the male and 1.8-fold change in the female rats when compared to the controls. In addition, for the cervical VN, in terms of the immunoreactivity of Kir3.1, there was 2.2-fold change in male and 2.4-fold change in female rats over thoracic VN, * *p* < 0.05, ** *p* < 0.05, and *** *p* < 0.05. All these fold changes are depicted in Figure 6B. The main reason for the investigation of the VN in two different regions is to enable the examination of the cervical VN–brainstem relationship and the thoracic VN–heart relationship. Because it is known that the main center of VN stimulation therapy is cervical VN, an increase in the immunoreactivity of it in terms of the Kir3.1 channel and M2 receptor compared to the thoracic VN indicates the evaluation of the brainstem and ACh-associated centers in epilepsy. After reflected in the results, a new ACh-related center might be offered to this core center as a candidate.

#### 3.3.3. Cardiac Tissue

**Figure 7:**
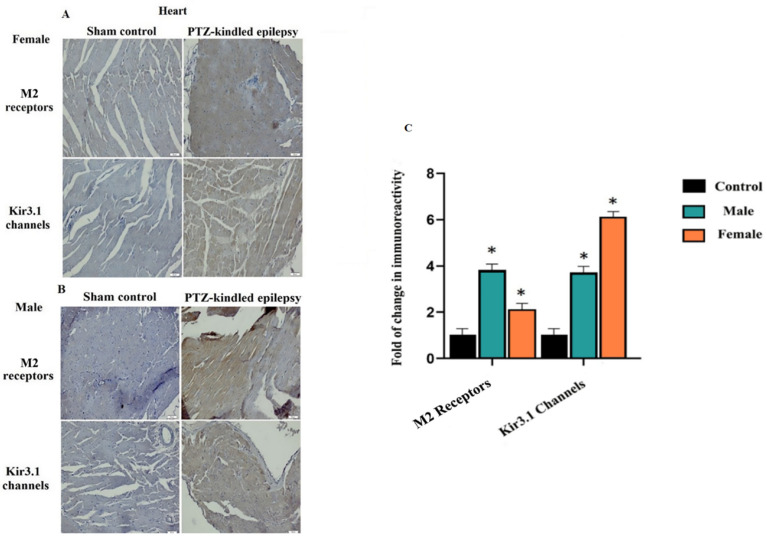
Examples of immunoreactivity for M2 receptors and Kir3.1 channel within the heart tissue (atria). Immunoreactivity was significantly increased for both ACh-associated centers and Kir3.1 channel, as demonstrated in the represented images. Results for female rats are demonstrated in section **A**, whereas for male rats they are demonstrated in section **B**. Pictures were taken at a magnification of ×200. Scale bar: 50 μm. Fold change in immunoreactivity for M2 receptors and Kir3.1 channel within the heart tissue (**C**). For female PTZ-kindled rats, there was a 2.1-fold change in M2 and a 6.1-fold change in the Kir3.1 channel when compared to the controls. On the other hand, for male PTZ-kindled rats, there was a 3.8-fold change in M2 receptors and a 3.7-fold change in the Kir3.1 channel when compared to the controls, * *p* < 0.05.

The immunoreactivity results of the M2 receptor and Kir3.1 ion channel in the cardiac tissue from both female and male rats are shown in Figure 7A,B, respectively. In both female and male rats, the areas stained with M2 receptors and Kir3.1 in the experimental groups significantly increased. For female PTZ-kindled rats there was a 2.1-fold change in the M2 receptors and a 6.1-fold change in the Kir3.1 channel when compared to the controls. On the other hand, for male PTZ-kindled rats, there was a 3.8-fold change in the M2 receptors and a 3.7-fold change in the Kir3.1 channel when compared to the controls, * *p* < 0.05, ** *p* < 0.05, and *** *p* < 0.05. All these fold changes are depicted in Figure 7C. The reason why the atrium was targeted is that the ACh-associated centers in the heart are located here. The immunoreactive increase in the Kir3.1 channel and M2 receptor in the atrium of the heart is consistent with the results of the VN and brainstem. This compliance describes the involvement of the ANS in terms of parasympathetic ACh-related centers.

## 4. Discussion

Generalized tonic–clonic seizures vary autonomic function during ictal, interictal, and postictal periods. All aspects of autonomous centers, including parasympathetic and sympathetic systems, might be affected. Autonomic involvement is the most prominent symptom of simple partial seizures but may not be recognized. Seizures typically activate sympathetic nerve activity and increase heart-rate, but parasympathetic activation or sympathetic inhibition could be dominant during partial seizures. Moreover, the seizure-induced cardiovascular function may contribute to SUDEP [31]. In this paper, the role of autonomic involvement in epilepsy was investigated for the first time in terms of the ACh neurotransmitter, which plays a key role in both parasympathetic and sympathetic activity.

ACh, one of the two most prevalent neurotransmitters released by ANS neurons, is secreted by cholinergic fibers. Accordingly, ACh binds to two types of cholinergic receptors. Nicotinic receptors are found in the cell bodies of all postganglionic neurons, both sympathetic and parasympathetic in the ANS ganglia [32]. The primary mechanism used by cholinergic synapses is enzymatic degradation. Acetylcholinesterase (AChE) is responsible for hydrolyzing ACh. It is known that AChE is distributed in nerve tissue such as the brainstem, cerebellum, peripheral and autonomic nervous systems [33]. ACh, released as a neural signal, spreads and acts on the cell membrane. As a result, the ACh receptor known as M2 undergoes a conformational change and the membrane releases relevant ions [34]. Based on the available literature, we speculated an ACh-based direction and investigated the Kir3.1 that directs ACh-activated K^+^ flow (I_KACh_) and the M2 receptor.

It was shown that Kir3.1 channels are expressed in the heart and are especially predominant in the atria [35]. In another study, it was concluded that Kir3.1 gives I_KACh_ properties that increase channel activity, while Kir3.4 channels did not contribute significantly to the I_KACh_ currents [36]. Therefore, in this study, only the Kir3.1 channel from the Kir family was focused. 

Brainstem–brain interactions have been shown to modulate the activity and neurotransmitter release of cortical and subcortical neurons and generate critical activity states by synchronizing the firing of larger neuron populations [37,38,39]. In a related study, it was determined that seizure activity induces pronounced autonomic activation and disrupts activity in the medullary respiratory centers, resulting in death from obstructive or central apnea [40]. It was shown in the study that respiratory problems are centered on the brainstem for the postictal period. It was found that VNS treatments caused an increase in basal neuronal release in the nuclei found in the brainstem for serotonin and norepinephrine [41]. However, there is still no pre-clinical study related to cardiac involvement in the brainstem. In epilepsy studies of the region known as the “lower brainstem”, clinical histopathological anomalies were reported in tissue samples taken from patients who died with a diagnosis of SUDEP [42]; however, no detailed information about the molecular mechanism has been reported. The relationship of this region with epilepsy has been revealed by clinical MRI studies and involvement at autonomic centers has been confirmed [43]. 

In our current study of the “lower brainstem” region, containing pons of the brainstem, we reported a significant increase in the immune reactivation of both the Kir3.1 channel and M2 receptors in the PTZ-kindled animals. The ACh-associated immunoreactive increase is likely to indicate that major cholinergic centers in the brainstem could become active in the postictal period and may both spread to the brain and affect the ANS via the VN. Thus, we aimed to investigate not only the parasympathetic effect but also the sympathetic effect.

Human VNs contain sympathetic nerve fibers and play a crucial role in the physiological effects reported with VNS. Molecular investigation of sympathetic nerve fibers in VNs may lead to a better understanding of the therapeutic mechanisms of VNS [44]. The left VN is known as the stimulation area for epilepsy in the treatment of VNS. Moreover, the left vagosympathetic trunk innervates the atrioventricular node in the dog and has not been reported to cause changes in heart rate [45] and there is very limited information about the cardiac effects during cervical VNS for epilepsy [46]. Accordingly, the fact that ACh released into the heart with left thoracic VN does not affect the associated centers, such as M2 receptors and Kir3.1, corresponds with the results we obtained. In contrast, the immunoreactive increase in the M2 receptor and Kir3.1 target we achieved in cervical VN is expected to be associated with epilepsy due to coding from the brainstem. Moreover, these increases may have emphasized a new center related to ACh in VNS. As is known, it is essential to understand the peripheral anatomy and physiology of the VN and central afferent projections to understand how VNS reduces or eliminates seizure activity. Accordingly, dividing the results into two, as both thoracic and cervical VN, made the interpretation robust.

Kir channels are expressed in both the heart and brain, have been shown to cause a change in epilepsy and may be responsible for the development of chronic epilepsy-induced cardiac arrhythmias [47]. Furthermore, cortical autonomic dysfunction resulting from seizure activity has been suggested to play a role, where it is thought that seizure activity might affect cardiac function since it changes the autonomic output centralized to the heart [1]. The increased immunoreactivity of Kir3.1 channel, especially in the “lower brainstem” region and cervical VN, highlighted the importance of an investigation into the M2 receptors and Kir3.1 channel in the cardiac tissue. This is because, in previous studies, it was determined by clinical studies that brainstem centers and cervical VN play a prominent role in cardiac involvement [48,49].

Significantly increases in immune reactivity in the M2 receptor and Kir3.1 channel obtained in cardiac tissue have shown that the ACh-related region is affected not only in the brainstem and its vicinity but also in another autonomic involvement center. These results speculate the involvement of autonomic centers as systems in terms of ACh in epilepsy, making these a further target for the investigation of other centers related to this neurotransmitter. In our epilepsy model of both the M2 receptor and Kir3.1 channel, the absence of functional research with a patch clamp appears to be a limitation of the study. The urgent investigation of norepinephrine, one of the two neurotransmitters released from the ANS neurons, with the same hypothesis might provide insights about another center for autonomic involvement.

ACh is the crucial neurotransmitter for the brainstem, VN and heart tissues. The main reason is that ACh has a parasympathetic effect by the release of the brainstem from the VN nuclei to the heart tissue [50]. In an earlier study, it was shown that heart rate and electrocardiogram (ECG)values increased during the ictal period [47]. Based on these facts, ACh-related centers are expected to be upregulated, reflecting a possible autonomic involvement. Upregulation in the M2 receptor reflects the fact that more ACh neurotransmitters might be attached to the ACh receptor. Similarly, upregulation in the Kir3.1 channel and increased immunoreactivity confirms this hypothesis in terms of ACh activation. Upregulation in the brainstem and in the heart [51] could be defined by excessive parasympathetic activation, and upregulation in the VN [52] by an excessive sympathetic effect. Thus, it is predicted that sympathetic and parasympathetic balance might be impaired in epilepsy with increases in ACh-related Kir3.1 channels and M2 receptors.

In conclusion, the current study, via utilizing an animal model of epilepsy, provides evidence that Ach-related immunoreactivity could be associated with the dysfunction of ANS accompanied by epilepsy.
